# Performance of 22 Rapid Lateral Flow Tests for SARS-CoV-2 Antigen Detection and Influence of “Variants of Concern”: Implications for Clinical Use

**DOI:** 10.1128/spectrum.01157-22

**Published:** 2022-07-12

**Authors:** Aurélie Gourgeon, Alexandre Soulier, Étienne Audureau, Souraya Khouider, Arnaud Galbin, Camille Langlois, Magali Bouvier-Alias, Christophe Rodriguez, Stéphane Chevaliez, Jean-Michel Pawlotsky, Slim Fourati

**Affiliations:** a Department of Virology, Henri Mondor Hospital, Assistance Publique-Hôpitaux de Paris, Université Paris-Est, Créteil, France; b INSERM U955, Institut Mondor de Recherche Biomédicale, Créteil, France; c Department of Public Health and CEpiA Team, Henri Mondor Hospital, Assistance Publique-Hôpitaux de Paris, Université Paris-Est, Creteil, France; Hôpital Saint-Louis

**Keywords:** lateral-flow tests, SARS CoV-2, variants of concern

## Abstract

Large-scale head-to-head assessment of the performance of lateral-flow tests (LFTs) for severe acute respiratory syndrome coronavirus 2 (SARS-CoV-2) antigen is required in the context of the continuous emergence of new viral variants. The aim of this study was to evaluate the performance of 22 rapid LFTs for the detection of SARS-CoV-2 antigens. The clinical performance of 22 LFTs was evaluated in 1,157 samples collected in the Greater Paris area. The 8 best-performing LFTs were further assessed for their ability to detect 4 variants of concern (VOC), including the alpha, beta, delta, and omicron (BA.1) variants. The specificity of SARS-CoV-2 LFTs was generally high (100% for 15 of them) but was insufficient (<75%) for 3 tests. Sensitivity of the LFTs varied from 30.0% to 79.7% compared to nucleic acid amplification testing (NAAT). Using a cycle threshold (*C_T_*) cutoff of ≤25, sensitivity of the assays ranged from 59.7% to 100%. The 8 best-performing assays had a sensitivity of ≥80% for the detection of the 4 VOC when the *C_T_* was ≤25. Falsely negative SARS-CoV-2 antigen LFT results were observed with omicron, due to the occurrence of low viral loads (*C_T_* > 30 in 32% of samples) during the two first days following symptom onset. Several LFTs exhibited satisfactory sensitivity and specificity, whereas a few others yielded an unacceptable proportion of false-positive results and/or lacked sensitivity. The sensitivity of the best-performing assays was not influenced by VOC, including alpha, beta, delta, and omicron variants. The ability of LFTs to detect the omicron variant could be reduced during the first days following symptom onset due to lower viral loads than with other variants.

**IMPORTANCE** The use of lateral-flow tests (LFTs) to detect SARS-CoV-2 has expanded worldwide. LFTs detect SARS-CoV-2 viral antigen and are less sensitive than nucleic acid amplification testing (NAAT). Their performance must be evaluated independently of the manufacturers. Our study assessed the performance of 22 SARS-CoV-2 antigen LFTs in large panels of well-characterized samples. The majority of LFTs tested exhibited satisfactory sensitivity and specificity, while some assays yielded unacceptable proportions of false-positive results, and others lacked sensitivity for samples containing large amounts of virus. The sensitivity of the best-performing assays did not vary according to the VOC, including the alpha, beta, delta, and omicron variants.

## INTRODUCTION

Severe acute respiratory syndrome coronavirus 2 (SARS-CoV-2) is the pathogen responsible for coronavirus disease 2019 (COVID-19) pandemic, which spread across the world at the end of 2019 and beginning of 2020 and has now become endemic worldwide, with regular epidemic waves. In many regions, mass testing in congregate settings remains an important component of the global strategy against SARS-CoV-2 infection, with the goal to identify and isolate infected individuals early enough to reduce disease transmission. The importance of mass testing has been reinforced by the emergence of viral variants, including some that have been classified as variants of concern (VOC) by the World Health Organization. Among them, the most recent variant, omicron, has been shown to be more transmissible than previous VOC, while its spread has been characterized by reduced effectiveness of public health measures and vaccine efficacy.

The reference method to diagnose SARS-CoV-2 infection is SARS-CoV-2 RNA detection in nasopharyngeal swabs (NPSs) by means of a nucleic acid amplification test (NAAT). NAATs use real-time reverse transcription-PCR (RT-PCR), real-time transcription-mediated amplification (TMA), or loop-mediated isothermal amplification (LAMP). NAAT disadvantages include the need for centralized laboratories, skillful operators, expensive instrumentation, sample batching, and biosafety measures. Furthermore, NAAT positivity can be a poor indicator of infectiousness, because patients can shed measurable amounts of viral RNA in the absence of infectious viruses. Since mid-2020, less expensive, easy-to-use, more rapid diagnostic tests have become commercially available. These tests are designed to detect the presence of antigens expressed by SARS-CoV-2 proteins produced during the viral life cycle and present in respiratory secretions. Two types of SARS-CoV-2 antigen tests are available: (i) laboratory-based enzyme immunoassays (EIA) and (ii) rapid lateral-flow tests (LFTs) intended for point-of-care use. The latter are easy to perform with limited training in various settings, such as during epidemic waves, before attending social gatherings, etc. In France, approximately 75% of daily COVID-19 diagnoses are currently based on SARS-CoV-2 antigen tests.

As of today, more than 200 different SARS-CoV-2 antigen LFTs exist on the market. Several studies have compared the performance of some LFTs with that of NAAT. They showed reduced analytical sensitivity of antigen tests compared to NAAT, particularly in asymptomatic individuals and in samples containing low SARS-CoV-2 RNA levels. However, these studies were generally based on small panels of samples and evaluated one or a small number of LFT antigen tests. Thus, large-scale independent head-to-head assessments of SARS-CoV-2 antigen LFTs are needed to guide their use in clinical practice and for public health interventions.

The aim of this study was to evaluate the performance of a large number (*n* = 22) of rapid, point-of-care LFTs for the direct detection of SARS-CoV-2 antigens. The study was split into two parts: (i) in the first part, which used 1,157 samples, the sensitivity of the 22 LFTs relative to NAAT was measured in samples collected in the Greater Paris area during the first or the second and third waves of the COVID-19 pandemic, while their specificity was assessed in frozen NPSs collected before the emergence of the pandemic; (ii) in the second part of the study, 8 LFTs identified as having good sensitivity and specificity in the first part of the study were assessed for their ability to detect 4 VOC, including the alpha, beta, delta, and omicron variants.

## RESULTS

### Study part 1: assessment of performance of 22 SARS-CoV-2 antigen LFTs. (i) Samples and design.

In the first part of the study, the performance of 22 SARS-CoV-2 antigen LFTs (listed with their technical specifications in Table S1 in the supplemental material) was evaluated relative to NAAT. Because sample volumes were not sufficient to assess more than 10 different LFTs, 3 panels of samples with comparable characteristics at inclusion were used. Sample selection from deidentified remnant swabs was randomly carried out according to stratification on cycle threshold (*C_T_*) values and, whenever possible, on the time of sampling relative to symptom onset in symptomatic patients. Their characteristics are shown in Table 1 and Fig. S1. A reference LFT that previously showed excellent sensitivity and specificity (COVID-VIRO antigen rapid test; AAZ-LMB, Boulogne-Billancourt, France [referred to here as AAZ]) (1) was used as a comparator across all experiments.

**TABLE 1:** Baseline characteristics of the 3 cohorts associated with the panels used in part 1 of the study, including 452 samples with positive SARS-CoV-2 RNA detection by RT-PCR and 526 samples not containing SARS-CoV-2 RNA

Cohort/panel and characteristic	No. (%) of samples		
Cohort/panel 1	SARS-CoV-2 RNA positive (*n* = 152)^*a*^	SARS-CoV-2 RNA negative (*n* = 150)^*b*^	SARS-CoV-2 RNA negative in transparent VTM (*n* = 101)^*c*^
Days from symptom onset			
≤3	56 (36.8)		
4–7	14 (9.2)		
8–11	7 (4.6)		
≥12	7 (4.6)		
Asymptomatic	20 (13.2)		
Unknown	48 (31.6)		
RT-PCR *C_T_* value			
≤20	21 (13.8)		
21–25	46 (30.3)		
26–30	38 (25.0)		
>30	47 (30.9)		
Other pathogens detected			
None		74 (49.3)	NT
Viral^*d*^		76 (50.7)	NT
Cohort/panel 2	SARS-CoV-2 RNA positive (*n* = 150)	SARS-CoV-2 RNA negative (*n* = 125)	
Days from symptom onset			
≤3	19 (12.7)		
4–7	18 (12.0)		
8–11	13 (8.7)		
≥12	5 (3.3)		
Asymptomatic	3 (2.0)		
Unknown	92 (61.3)		
RT-PCR *C_T_* value			
≤20	21 (14.0)		
21–25	46 (30.7)		
26–30	38 (25.3)		
>30	45 (30.0)		
Other pathogens detected			
None		100 (80.0)	
Viral^*e*^		25 (20.0)	
Cohort/panel 3	SARS-CoV-2 RNA positive (*n* = 150)	SARS-CoV-2 RNA negative (*n* = 150)	
Days from symptom onset			
≤3	25 (16.7)		
4–7	22 (14.8)		
8–11	20 (13.3)		
≥12	5 (3.3)		
Asymptomatic	1 (0.6)		
Unknown	77 (51.3)		
RT-PCR *C_T_* value			
≤20	21 (14.0)		
21–25	46 (30.7)		
26–30	32 (21.3)		
>30	51 (34.0)		
Other pathogens detected			
None		124 (82.7)	
Viral^*e*^		26 (17.3)	

a Sofia and Wantai LFTs could be evaluated with only a subset of this cohort (*n* = 133 and *n* = 118, respectively), due to possible interference between VTM and automatic immunofluorescence reading.

b This panel could not be used to assess the specificity of Sofia and Wantai LFTs, due to possible interference between VTM and automatic immunofluorescence reading.

c This additional panel was collected on transparent VTM to assess the specificity of Sofia and Wantai LFTs to avoid interference with automatic immunofluorescence. NT, not tested.

d Coronaviruses other than SARS-CoV-2 were detected in 12 patients, including HKU1 (*n* = 3), NL63 (*n* = 3), 229E (*n* = 3), and OC43 (*n* = 3).

e Coronaviruses other than SARS-CoV-2 were detected in 8 patients, including HKU1 (*n* = 2), NL63 (*n* = 2), 229E (*n* = 2), and OC43 (*n* = 2).

### (ii) Rate of success.

The rate of success of the 22 LFTs was high, being between 96% and 100% (Table S2). The result was invalid (absence of the control bar) in 32 of the 978 samples tested. Twenty invalid results were obtained in SARS-CoV-2 RNA-positive samples (GenBody, *n* = 8; GenSure, *n* = 2; AMP, *n* = 1; QuickProfile, *n* = 1; Novel, *n* = 2; Toda Pharma, *n* = 2; Sofia, *n* = 1; Fasual Care, *n* = 1; R-Biopharm, *n* = 1; and Orgentec, *n* = 1 [for the full names of tests, see Table 2]), and the remaining 12 were in SARS-CoV-2 RNA-negative samples (GenBody, *n* = 4; Nadal, *n* = 4; AMP, *n* = 1; Novel, *n* = 1; Fasual Care, *n* = 1; and Humasis, *n* = 1).

### (iii) Specificity.

To assess SARS-CoV-2 antigen test specificity, 425 SARS-CoV-2 RNA-negative samples collected between April and August 2019, i.e., before the emergence of SARS-CoV-2, in patients and health care workers (HCWs) presenting with a suspicion of respiratory infection were used (Table 1). The reasons for using pre-SARS-CoV-2 pandemic samples for specificity testing were the need for true negatives and the availability of such well-characterized samples (including some containing other respiratory viruses) stored at −80°C. Table S3 lists respiratory viruses found in some of the SARS-CoV-2 RNA-negative samples from the 3 panels. An additional panel of 101 NPSs collected in a transparent viral transport medium (VTM) between October and November 2020 from patients and HCWs with negative SARS-CoV-2 RNA detection was used to assess the specificity of two assays that could not be used with the VTM from panel 1 (Table 1). Indeed, the presence of the phenol red pH indicator in the VTM has been reported to be associated with false-positive results with the 2 automatic-reading assays (Sofia and Wantai).

**FIG 1:**
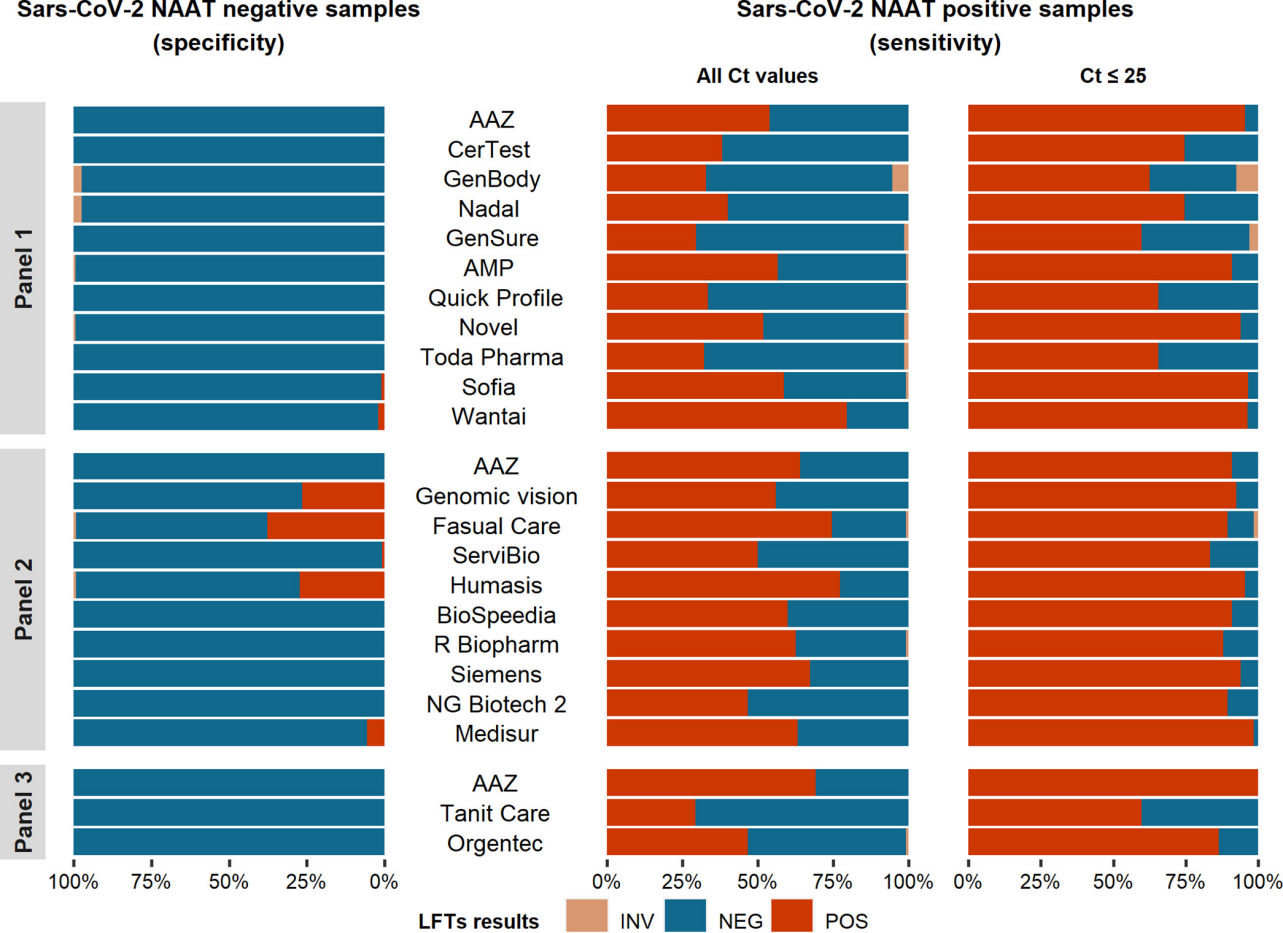
Specificities and sensitivities of the 22 SARS-CoV-2 antigen LFTs tested in part 1 of this study. Sensitivity was calculated relative to the presence of SARS-CoV-2 RNA at any *C_T_* level in RT-PCR and for *C_T_* values ≤25. INV, invalid; NEG, negative; POS, positive.

As shown in Table 2 and Fig. 1, the specificity of the 22 LFTs relative to NAAT ranged from 62.4% to 100%. The specificities of 3 LFTs were considered insufficient, including those of Fasual Care (62.4%; 95% confidence interval [95% CI], 53.3% to 70.9%), Humasis (72.8%; 95% CI, 64.1% to 80.4%) and Genomic Vision (73.6%; 95% CI, 65.0% to 81.1%). None of the samples falsely positive with Fasual Care (*n* = 47) or Humasis (*n* = 34) contained another respiratory virus, whereas 2 of the 33 samples that were falsely positive with Genomic Vision contained another respiratory virus (human influenza A virus in one case, parainfluenza 4 virus in the other case). One LFT (Medisur) had intermediate specificity (94.4%; 95% CI, 88.8% to 97.7%). Three other LFTs, including the two assays with automated reading (Sofia and Wantai) and a visual reading test (Servibio), had an acceptable specificity, ≥97.0% (as per WHO criteria), displaying occasional false-positive results. The remaining 15 tests had 100% specificity (Table 2 and Fig. 1).

**TABLE 2:** Specificity relative to NAAT of the 22 SARS-CoV-2 antigen LFTs

Panel	Test	Manufacturer	Abbreviation	Specificity (95% CI) (%)
1	COVID-VIRO antigen rapid test	AAZ	AAZ	100 (97.6–100)
	CerTest SARS-CoV-2 card test	CerTest Biotec	CerTest	100 (97.6–100)
	GenBody COVID-19 Ag	GenBody Inc.	GenBody	100 (97.5–100)
	Nadal COVID-19 Ag test	Nal von Minden	Nadal	100 (97.5–100)
	Rapid test antigen GenSure COVID-19	GenSure	GenSure	100 (97.6–100)
	AMP rapid test SARS-CoV-2 Ag	AMP Diagnostics	AMP	100 (97.6–100)
	QuickProfile COVID-19 antigen test	LumiQuick Diagnostics	QuickProfile	100 (97.6–100)
	Novel coronavirus (COVID-19) antigen test kit	Medakit	Novel	100 (97.6–100)
	Toda Coronadiag Ag	Toda Pharma	Toda Pharma	100 (97.6–100)
	Sofia SARS antigen FIA	Quidel	Sofia	99 (94.6–100)
	Wantai SARS-CoV-2 Ag rapid test	Eurobio Scientific	Wantai	98 (93.0–99.8)

2	COVID-VIRO antigen rapid test	AAZ	AAZ	100 (97.1–100)
	SARS-CoV-2 antigen rapid detection kit	Genomic Vision	Genomic Vision	73.6 (65.0–81.1)
	RealityTech antigen test COVID 19	Fasual Care	Fasual Care	62.4 (53.3–70.9)
	COVID-19 antigen rapid test	Servibio	Servibio	99.2 (95.7–100)
	Humasis one-step COVID-19 Ag test	Eurobio Scientific	Humasis	72.8 (64.1–80.4)
	BSD-0500333-25- COVID19 speed antigen test	Biospeedia	Biospeedia	100 (97.1–100)
	SARS-CoV-2 spike colloidal gold chromatographic assay	R-Biopharm	R-Biopharm	100 (97.1–100)
	Test antigénique rapide Clinitest COVID-19	Siemens Healthcare	Siemens	100 (97.1–100)
	NG-Test COVID19	NG-Biotech	NG Biotech 2	100 (97.1–100)
	Indicaid COVID-19 rapid antigen test	Medisur	Medisur	94.4 (88.8–97.7)

3	COVID-VIRO antigen rapid test	AAZ	AAZ	100 (97.6–100)
	PCL test COVID Ag	Tanit Care	Tanit Care	100 (97.6–100)
	Biosensor standard F COVID-19 Ag FIA	Orgentec	Orgentec	100 (97.6–100)

### (iv) Sensitivity.

To assess antigen test sensitivity, 452 SARS-CoV-2 RNA-positive samples collected between March and November 2020 (first epidemic wave), then between January 2021 and January 2022 (second to fifth epidemic waves) in SARS-CoV-2-infected patients and HCWs with or without COVID-19 symptoms were used (Table 1). All samples were stored at −80°C. Although LFT assays have not been designed for use with frozen samples, freeze-thaw procedures have been shown to minimally affect LFT assay sensitivity, particularly for *C_T_* values of ≤30 (2, 3).

**FIG 2:**
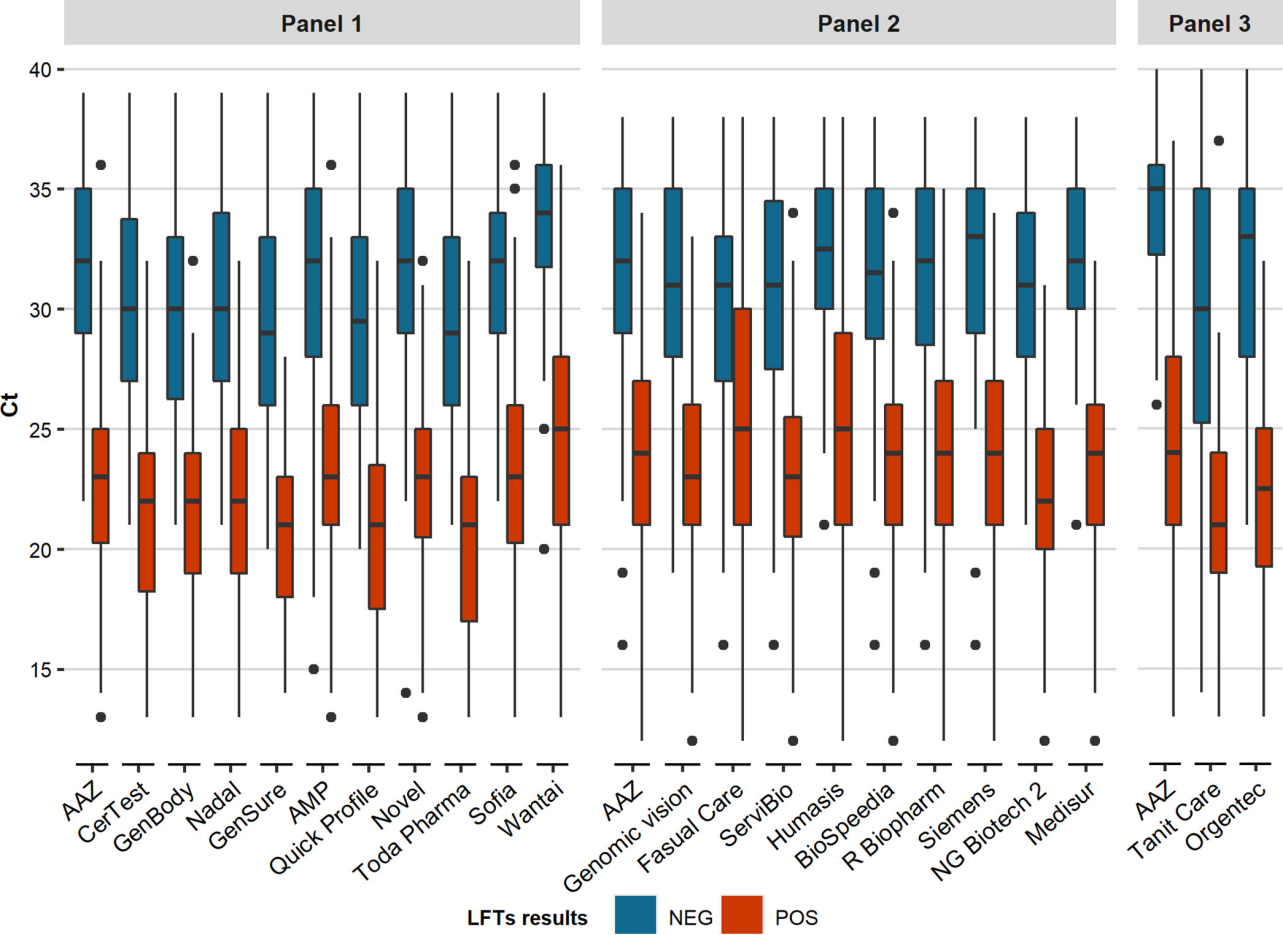
Distribution of LFT results in SARS-CoV-2 RNA-positive samples according to *C_T_* values in NAAT. NEG, negative LFT result; POS, positive LFT result.

As shown in Fig. 1 and Table 3, the global LFT sensitivities relative to NAAT ranged from 30.0% for GenSure (95% CI, 22.8% to 38.0%) to 79.7% for Wantai (95% CI, 71.3% to 86.5%). The sensitivities of the 22 SARS-CoV-2 antigen LFTs in samples with *C_T_* values of ≤25 in NAAT ranged from 59.7% for Tanit Care (95% CI, 47.0% to 71.5%) to 100% for AAZ (95% CI, 94.6% to 100%). Using the *C_T_* cutoff of 25, 15 of the 22 LFTs were considered to have an acceptable sensitivity (>80%). They included AAZ, AMP, Novel, Sofia, Wantai, Genomic Vision, Fasual Care, Servibio, Humasis, Biospeedia, R-Biopharm, Siemens, NG Biotech 2, Medisur, and Orgentec (Fig. 2 and Table 3).

**TABLE 3:** Sensitivities of SARS-CoV-2 antigen LFTs according to the number of days from symptom onset and the viral load, as assessed by the *C_T_* value in RT-PCR

Cohort and category	Sensitivity (95% CI) (%) of test					
Cohort 1 (1)	AAZ	CerTest	GenBody	Nadal	GenSure	AMP
Days from symptom onset						
≤7	70.0 (57.9–80.4)	50.0 (37.8–62.2)	47.7 (35.1–60.5)	52.9 (40.6–64.9)	43.5 (31.6–56.0)	68.1 (55.8–78.8)
8–11	42.9 (9.9–81.6)	14.3 (0.4–57.9)	14.3 (0.4–57.9)	28.6 (3.4–71.0)	14.3 (0.4–57.9)	42.9 (9.9–81.6)
≥12	14.3 (0.4–57.9)	14.3 (0.4–57.9)	14.3 (0.4–57.9)	14.3 (0.4–57.9)	0 (0–45.9)	14.3 (0.4–57.9)
*C_T_* value						
≤20	100 (83.9–100)	100 (83.9–100)	100 (80.5–100)	100 (83.9–100)	94.7 (74.0–99.9)	90.5 (69.6–98.8)
21–25	93.5 (82.1–98.6)	63.0 (47.5–76.8)	55.6 (40–70.4)	63.0 (47.5–76.8)	47.8 (32.9–63.1)	91.3 (79.2–97.6)
26–30	39.5 (24.0–56.6)	18.4 (7.7–34.3)	18.9 (8.0–35.2)	23.7 (11.4–40.2)	13.2 (4.4–28.1)	48.6 (31.9–65.6)
>30	6.4 (1.3–17.5)	2.1 (0.1–11.3)	2.2 (0.1–11.8)	4.3 (0.5–14.5)	0 (0–7.5)	14.9 (6.2–28.3)
≤25	95.5 (87.5–99.1)	74.6 (62.5–84.5)	67.6 (54.7–79.1)	74.6 (62.5–84.5)	61.5 (48.6–73.3)	91.0 (81.5–96.6)
≤30	75.2 (65.9–83.1)	54.3 (44.3–64.0)	49.5 (39.3–59.7)	56.2 (46.2–65.9)	43.7 (33.9–53.8)	76.0 (66.6–83.8)
Any	53.9 (45.7–62.1)	38.2 (30.4–46.4)	34.7 (27.0–43.1)	40.1 (32.3–48.4)	30.0 (22.8–38)	57 (48.7–65)

Cohort 1 (2)	QuickProfile	Novel	Toda Pharma	Sofia	Wantai	
Days from symptom onset						
≤7	46.4 (34.3–58.8)	68.6 (56.4–79.1)	47.4 (35.1–59.4)	80.0 (67–89.6)	83.9 (71.7–92.4)	
8–11	0 (0–41.0)	33.3 (4.3–77.7)	0 (0–41.0)	28.6 (3.7–71.0)	60.0 (14.7–94.7)	
≥12	14.3 (0.4–57.9)	14.3 (0.4–57.9)	14.3 (0.4–57.9)	20.0 (0.5–71.6)	25.0 (0.6–80.6)	
*C_T_* value						
≤20	95.2 (76.2–99.9)	95.2 (76.2 -99.9)	100 (83.9–100)	100 (83.2–100)	95.0 (75.1–99.9)	
21–25	52.2 (36.9–67.1)	93.5 (82.1–98.6)	50.0 (34.9–65.1	94.7 (82.3–99.4)	97.3 (85.8–100)	
26–30	13.5 (4.5–28.8)	37.8 (22.5–55.2)	10.8 (3.0–25.4)	45.5 (28.1–63.6)	89.3 (71.8–97.7)	
>30	4.3 (0.5–14.5)	4.3 (0.5–14.8)	2.2 (0.1–11.5)	17.1 (7.2–32.1)	42.4 (25.5–60.8)	
≤25	65.7 (53.1–76.8)	94.0 (85.4–98.3)	65.7 (53.1–76.8)	96.6 (88.1–99.6)	96.5 (87.9–99.6)	
≤30	47.1 (37.2–57.2)	74.0 (64.5–82.1)	46.2 (36.3–56.2)	78.0 (68.1–86.0)	94.1 (86.8–98.1)	
Any	33.7 (26.3–42)	52.7 (44.4–60.9)	32.7 (25.2–40.8)	59.1 (50.2–67.6)	79.7 (71.3–86.5)	

Cohort 2 (1)	AAZ	Genomic Vision	Fasual Care	Servibio	Humasis	
Days from symptom onset						
≤7	62.2 (44.8–77.5)	48.6 (31.9–65.6)	73.0 (55.9–86.2)	51.4 (34.4–68.1)	86.5 (71.2–95.5)	
8–11	30.8 (9.1–61.4)	30.8 (9.1–61.4)	38.5 (13.9–68.4)	7.7 (0.2–36.0)	38.5 (13.9–68.4)	
≥12	40.0 (5.3–85.3)	20.0 (0.5–71.6)	80.0 (28.4–99.5)	0 (0–52.2)	40.0 (5.3–85.3)	
*C_T_* value						
≤20	90.5 (69.6–98.8)	90.5 (69.6–98.8)	90.5 (69.6–98.8)	90.5 (69.6–98.8)	100 (83.9–100)	
21–25	91.3 (79.2–97.6)	93.5 (82.1–98.6)	91.1 (78.8–97.5)	80.4 (66.1–90.6)	93.5 (82.1–98.6)	
26–30	63.2 (46–78.2)	36.8 (21.8–54)	71.1 (54.1–84.6)	34.2 (19.6–51.5)	78.9 (62.7–90.4)	
>30	24.4 (12.9–39.5)	17.8 (8–32.1)	55.6 (40–70.4)	13.3 (5.1–26.8)	48.9 (33.7–64.2)	
≤25	91.0 (81.5–96.6)	92.5 (83.4–97.5)	90.9 (81.3–96.6)	83.6 (72.5–91.5)	95.5 (87.5–99.1)	
≤30	81.0 (72.1–88.0)	72.4 (62.8–80.7)	83.7 (75.1–90.2)	65.7 (55.8–74.7)	89.5 (82.0–94.7)	
Any	64.0 (55.8–71.7)	56.0 (47.7–64.1)	75.2 (67.4–81.9)	50.0 (41.7–58.3)	75.3 (67.6–82.0)	

Cohort 2 (2)	Biospeedia	R-Biopharm	Siemens	NG Biotech 2	Medisur	
Days from symptom onset						
≤7	54.0 (36.9–70.5)	67.6 (50.2–82)	70.3 (53.0–84.1)	40.5 (24.8–57.9)	62.2 (44.8–77.5)	
8–11	30.8 (9.1–61.4)	23.1 (5.0–53.8)	38.5 (13.9–68.4)	15.4 (1.9–45.4)	30.8 (9.1–61.4)	
≥12	20.0 (0.5–71.6)	20.0 (0.5–71.6)	40.0 (5.3–85.3)	0 (0–52.2)	20.0 (0.5–71.6)	
*C_T_* value						
≤20	90.5 (69.6–98.8)	90.5 (69.6–98.8)	90.5 (69.6–98.8)	100 (83.9–100)	100 (83.9 -100)	
21–25	91.3 (79.2–97.6)	87.0 (73.7–95.11)	95.7 (85.2–99.5)	84.8 (71.1–93.7)	97.8 (88.5–99.9)	
26–30	50.0 (33.74–66.6)	60.5 (43.4–76)	68.4 (51.3–82.5)	23.7 (11.4–40.2)	60.5 (43.4–76)	
>30	22.2 (11.2–37.1)	27.3 (14.9–42.8)	26.7 (14.6–41.9)	2.2 (0.1–11.8)	13.3 (5.1–26.8)	
≤25	91.0 (81.5–96.6)	88.1 (77.8–94.7)	94.0 (85.4–98.3)	89.6 (79.7–95.7)	98.5 (92–100)	
≤30	76.2 (66.9–84.0)	78.1 (69.0–85.6)	84.8 (76.4–91.0)	65.7 (55.8–74.7)	84.8 (76.4–91.0)	
Any	60.0 (51.7–67.9)	63.1 (54.8–70.8)	67.3 (59.2–74.8)	46.7 (38.5–55)	63.3 (55.1–71)	

Cohort 3	AAZ	Tanit Care	Orgentec			
Days from symptom onset						
≤7	66.0 (50.7–79.1)	23.5 (12.8–37.5)	46.8 (32.1–61.9)			
8–11	50.0 (27.2–72.8)	0 (0–16.8)	10.5 (1.3–33.1)			
≥12	40.0 (5.3–85.3)	20.0 (0.5–71.6)	20.0 (0.5–71.6)			
*C_T_* value						
≤20	100 (83.9–100)	90.5 (69.6–98.8)	100 (83.9–100)			
21–25	100 (92.3–100)	45.7 (30.9–61.0)	80.4 (66.1–90.6)			
26–30	81.3 (63.6–92.8)	9.4 (2.0–25.0)	32.3 (16.7–51.4)			
>30	21.6 (11.3–35.3)	2.0 (0–10.4)	3.9 (4.8–13.4)			
≤25	100 (94.6–100)	59.7 (47.0–71.5)	86.6 (76.0–93.7)			
≤30	93.9 (87.3–97.7)	43.4 (33.5–53.8)	69.4 (59.3–78.3)			
Any	69.3 (61.3–76.6)	29.3 (22.2–37.3)	47 (38.8–55.3)			

### Study part 2: assessment of performance for VOC detection of 8 SARS-CoV-2 antigen LFTs selected in part 1.

In the second part of the study, 8 SARS-CoV-2 antigen LFTs with a global specificity of ≥97% and a global sensitivity of ≥80% (minimum performance required by international recommendations) (4, 5) were tested for their ability to detect VOC that have been dominant over the successive epidemic waves in France since the end of 2020. The LFTs tested included AAZ, AMP, Novel, Biospeedia, R-Biopharm, Siemens, Abbott, and Biosynex (Table S1).

The SARS-CoV-2 VOC tested were the alpha, beta, delta, and omicron (BA.1) variants. The panel included 179 samples collected between January 2021 and January 2022 in which the infecting VOC had been identified by means of full-length genome sequence analysis using next-generation sequencing (alpha, *n* = 54; beta, *n* = 21; delta, *n* = 54; omicron, *n* = 50). The characteristics of the panel are shown in Table 4.

**TABLE 4:** Characteristics of panel 4, made up of 179 samples containing different VOC, as assessed by full-length SARS-CoV-2 genome sequencing, tested in part 2 of the study

RT-PCR *C_T_* value	No. (%) of samples containing variant			
	Alpha (*n* = 54)	Beta (*n* = 21)	Delta (*n* = 54)	Omicron (*n* = 50)
≤20	32 (59.3)	3 (14.3)	34 (63.0)	11 (22.0)
21–25	16 (29.6)	12 (57.1)	13 (24.1)	14 (28.0)
26–30	4 (7.4)	6 (28.6)	7 (13.0)	13 (26.0)
>30	2 (3.7)	0 (0)	0 (0)	12 (24.0)
Minimum (C_*T*_ value)	10	13	14	16
Maximum (C_*T*_ value)	40	29	30	37

**FIG 3:**
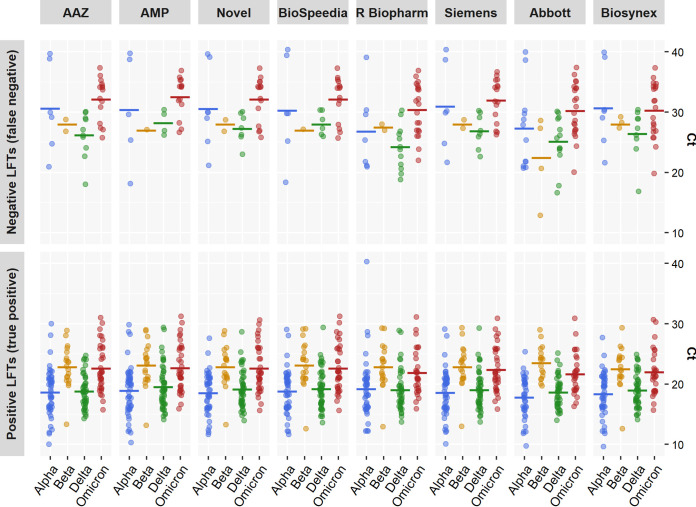
Distribution of *C_T_* values in samples found to be either negative (NEG) or positive (POS) in 8 SARS-CoV-2 antigen LFTs tested with the alpha, beta, delta, and omicron (BA.1) VOC.

As shown in Table 5 and Fig. 3, the global sensitivities of the 8 LFTs tested ranged from 77.4% (95% CI, 63.8% to 87.7%) with Abbott to 90.7% (95% CI, 79.7% to 96.9%) with AMP for the alpha, beta, and delta variants. Sensitivity for the detection of the omicron variant ranged from 56.0% (95% CI, 41.3% to 70.0%) to 70.0% (95% CI, 55.4% to 82.1%) for AAZ, AMP, Novel, and Biospeedia. As shown in Table 5, using the *C_T_* cutoff of 25, all of the 8 LFTs tested had a sensitivity of ≥80%, a threshold required by international recommendations.

**TABLE 5:** Sensitivities of SARS-CoV-2 antigen LFTs relative to NAAT for VOC detection according to the viral load, as assessed by the *C_T_* value in RT-PCR

Variant	*C_T_* value	Sensitivity (95% CI) (%) of test							
		AAZ	AMP	Novel	Biospeedia	R-Biopharm	Siemens	Abbott	Biosynex
Alpha	≤20	100 (89.1–100)	96.9 (83.8–99.9)	100 (88.8–100)	96.8 (83.3–99.9)	100 (88.1–100)	100 (89.1–100)	100 (89.1–100)	100 (89.1–100)
	21–25	87.5 (61.7–98.4)	93.8 (69.8–99.8)	86.7 (59.5–98.3)	93.3 (68.1–99.8)	73.3 (44.9–92.2)	87.5 (61.7–98.4)	60.0 (32.3–83.7)	87.5 (61.7–98.4)
	26–30	50.0 (6.8–93.2)	75.0 (19.4–99.4)	25.0 (0.6–80.6)	50.0 (6.8–93.2)	50.0 (6.8–93.2)	50.0 (6.8–93.2)	0 (0–60.2)	25.0 (0.6–80.6)
	>30	0 (0–84.2)	0 (0–84.2)	0 (0–84.2)	0 (0–84.2)	50.0 (1.3–98.7)	0 (0–84.2)	0 (0–84.2)	0 (0–84.2)
	≤25	95.8 (85.7–99.5)	95.8 (85.7–99.5)	95.7 (85.2–99.5)	95.7 (85.2–99.5)	90.9 (78.3–97.5)	95.8 (85.7–99.5)	87.2 (74.3–95.2)	95.8 (85.7–99.5)
	≤30	92.3 (81.5–97.9)	94.2 (84.1–98.8)	90.0 (78.2–96.7)	92.0 (80.8–97.8)	87.5 (74.8–95.3)	92.3 (81.5–97.9)	80.4 (66.9–90.2)	90.4 (79.0–96.8)
	Any	88.9 (77.4–95.8)	90.7 (79.7–96.9)	86.5 (74.2–94.4)	88.5 (76.6–95.6)	86.0 (73.3–94.2)	88.9 (77.4–95.8)	77.4 (63.8–87.7)	87.0 (75.1–94.6)

Beta	≤ 20	100 (29.2–100)	100 (29.2–100)	100 (29.2–100)	100 (29.2–100)	100 (29.2–100)	100 (29.2–100)	66.7 (9.4–99.2)	100 (29.2–100)
	21–25	100 (73.5–100)	100 (73.5–100)	100 (73.5–100)	100 (73.5–100)	100 (73.5–100)	100 (73.5–100)	91.7 (61.5–99.8)	100 (73.5–100)
	26–30	66.7 (22.3–95.7)	83.3 (35.9–99.6)	66.7 (22.3–95.7)	83.3 (35.9–99.6)	66.7 (22.3–95.7)	66.7 (22.3–95.7)	66.7 (22.3–95.7)	50.0 (11.8–88.2)
	>30								
	≤25	100 (78.2–100)	100 (78.2–100)	100 (78.2–100)	100 (78.2–100)	100 (78.2–100)	100 (78.2–100)	86.7 (59.5–98.3)	100 (78.2–100)
	≤30	90.5 (69.6–98.8)	95.2 (76.2–99.9)	90.5 (69.6–98.8)	95.2 (76.2–99.9)	90.5 (69.6–98.8)	90.5 (69.6–98.8)	81.0 (58.1–94.6)	85.7 (63.7–97.0)
	Any	88.9 (77.4–95.8)	90.7 (79.7–96.9)	86.5 (74.2–94.4)	88.5 (76.6–95.6)	86.0 (73.3–94.2)	88.9 (77.4–95.4)	77.4 (63.8–87.7)	87.0 (75.1–94.6)

Delta	≤20	97.1 (84.7–99.9)	100 (89.7–100)	100 (89.7–100)	100 (89.7–100)	94.1 (80.3–99.3)	100 (89.7–100)	94.1 (80.3–99.3)	97.1 (84.7–99.9)
	21–25	84.6 (54.6–98.1)	100 (75.3–100)	92.3 (64.0–99.8)	100 (75.3–100)	61.5 (31.6–86.1)	84.6 (54.6–98.1)	69.2 (38.6–0.9)	92.3 (64.0–99.8)
	26–30	0 (0–41)	42.9 (9.9–81.6)	14.3 (0.4–57.9)	14.3 (0.4–57.9)	28.6 (3.7–71.0)	14.3 (0.4–57.9)	0 (0–41.0)	0 (0–41.0)
	>30								
	≤25	93.6 (82.5–98.7)	100 (92.5–100)	97.9 (88.7–99.9)	100 (92.5–100)	85.1 (71.7–93.8)	95.7 (85.5–99.5)	87.2 (74.3–95.2)	95.7 (85.5–99.5)
	≤30	81.5 (68.6–90.7)	92.6 (82.1–97.9)	87.0 (75.1–94.6)	88.9 (77.4–95.8)	77.8 (64.4–88.0)	85.2 (72.9–93.4)	75.9 (62.4–86.5)	83.3 (70.7–92.1)
	Any	88.9 (77.4–95.8)	90.7 (79.7–96.9)	86.5 (74.2–94.4)	88.5 (76.6–95.6)	86.0 (73.3–94.2)	88.9 (77.4–95.8)	77.4 (63.8–97.7)	87.0 (75.1–94.6)

Omicron (BA.1)	≤20	100 (71.5–100)	100 (71.5–100)	100 (71.5–100)	100 (71.5–100)	100 (71.5–100)	100 (71.5–100)	90.9 (58.7–99.8)	90.9 (58.7–99.8)
	21–25	100 (76.8–100)	100 (76.8–100)	100 (76.8–100)	100 (76.8–100)	85.7 (57.2–98.2)	100 (76.8–100)	92.9 (66.1–99.8)	92.9 (66.1–99.8)
	26–30	69.2 (38.6–90.9)	76.9 (46.2–95.0)	69.2 (38.6–90.9)	69.2 (38.6–90.9)	38.5 (13.9–68.4)	61.5 (31.6–86.1)	30.8 (9.1–61.4)	38.5 (13.9–68.4)
	>30	8.3 (0.2–38.5)	8.3 (0.2–38.5)	8.3 (0.2–38.5)	8.3 (0.2–38.5)	8.3 (13.9–68.4)	8.3 (0.2–38.5)	8.3 (0.2–38.5)	8.3 (0.2–38.5)
	≤25	100 (86.3–100)	100 (86.3–100)	100 (86.3–100)	100 (86.3–100)	92.0 (74.0–99.0)	100 (86.3–100)	92.0 (74.0–99.0)	92.0 (74.0–99.0)
	≤30	89.5 (75.2–97.1)	92.1 (78.6–98.3)	89.5 (75.2–97.1)	89.5 (75.2–97.1)	73.7 (56.9–86.6)	86.8 (71.9–95.6)	71.1 (54.1–84.6)	73.7 (56.9–86.6)
	Any	70.0 (55.4–82.1)	70.0 (55.4–82.1)	70.0 (55.4–82.1)	70.0 (55.4–82.1)	58.0 (43.2–71.8)	68.0 (53.3–80.5)	56 (41.3–70.0)	58.0 (43.2–71.8)

**FIG 4:**
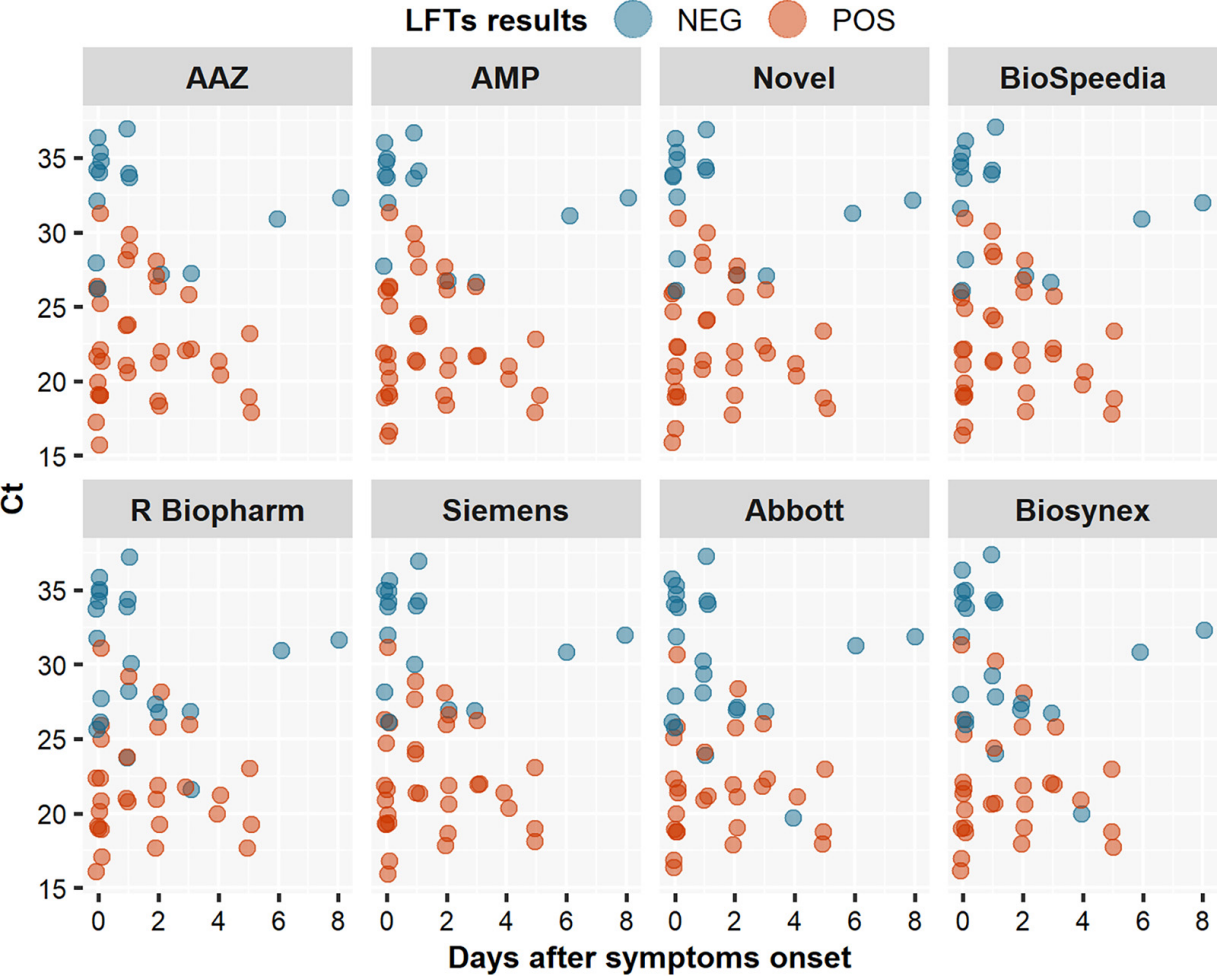
Distribution of *C_T_* values in samples found to be either negative (NEG) or positive (POS) in 8 SARS-CoV-2 antigen LFTs tested with the omicron variant (BA.1), according to the date after symptom onset.

The ability of the 8 LFTs to detect each VOC was evaluated using a bootstrap approach comparing their respective sensitivities to those for detection of the ancestral (Wuhan) SARS-CoV-2 strain. No difference in sensitivity was found for any of the 8 LFTs when VOC and the ancestral strain were compared, as shown by the following mean *P* values for the alpha, beta, delta, and omicron (BA.1) variants, respectively: for AAZ, 0.60, 0.65, 0.32, and 0.56; for Abbott, 0.52, 0.59, 0.47, and 0.54; for AMP, 0.50, 0.41, 0.42, and 0.42; for Biospeedia, 0.56, 0.42, 0.55, and 0.51; for Biosynex, 0.56, 0.63, 0.55, and 0.53; for Novel, 0.61, 0.50, 0.60, and 0.38; for R-Biopharm, 0.57, 0.54, 0.43, and 0.45; and for Siemens, 0.59, 0.67, 0.53, and 0.54 (Fig. 3). However, falsely negative SARS-CoV-2 antigen LFT results were observed with the omicron variant, due to low viral loads (*C_T_* > 30 in 32% [10/31] of NPSs) during the first days following symptom onset (Fig. 4).

## DISCUSSION

Large-scale SARS-CoV-2 testing is a key weapon in the fight against COVID-19. Testing can be used for diagnosis of infected patients, global surveillance, and/or targeted screening, with the goal of reducing community spread of the virus. Thus, achieving high testing capacities with easy-to-perform and inexpensive tests has become a priority. Solutions rely on the use of rapid diagnostic tests that detect SARS-CoV-2 antigens in nasopharyngeal, oropharyngeal, or nasal swabs. The use of SARS-CoV-2 antigen LFTs has been associated with interruptions of transmission if coupled with rapid isolation and cohorting of the most infectious cases and their close contacts (5, 6). In addition, SARS-CoV-2 antigen LFTs can be used for self-testing, a complement to health system-based testing by trained providers that offers many advantages, such as earlier, increased access to testing and care.

SARS-CoV-2 antigen tests have been reported to be less sensitive than NAATs in detecting the presence of the virus. The use of SARS-CoV-2 antigen LFTs for large-scale testing requires that their clinical performance be well known. Thus, there is a need for continuing independent postmarketing assessments of commercial antigen tests. Several studies evaluating the sensitivity and specificity of different SARS-CoV-2 antigen LFTs relative to RT-PCR have been published. However, the scope of brands evaluated was limited and the number of samples tested was relatively small, often restricted to symptomatic populations (7, 8). Interestingly, when individuals without symptoms were tested, higher proportions of subjects who are infectious appear to be missed (9).

Despite its high incidence during epidemic waves, COVID-19 infection has a low prevalence in the general population. Thus, high specificity (>97%) of the assays is required to avoid too-frequent false-positive results (4, 5). In our study, the majority of LFTs tested had very high specificity, regardless of the presence or absence of other respiratory viruses in the samples, suggesting a lack of cross-reactivity, including with endemic coronaviruses.

Sensitivity of the tests is crucial, more specifically their ability to diagnose infection in patients with viral loads associated with positive viral cultures, who are the most likely to transmit infection (RT-PCR *C_T_* < 25). Indeed, it has been shown that, despite a significantly lower analytical sensitivity than NAATs, SARS-CoV-2 antigen LFTs have the capacity to diagnose the majority of contagious cases (10, 11). As per WHO recommendations, LFT sensitivity should reach a minimum of 80% relative to nucleic acid testing. In a recent Cochrane systematic review of antigen LFT performance, the overall sensitivity reached 94.5% in samples containing high viral loads (*C_T_* ≤ 25), whereas it was only 40.7% in samples containing lower viral loads (7).

In the present study, we assessed the performance of a large number of different SARS-CoV-2 antigen LFTs with 4 large panels of samples, including RNA-positive and -negative ones and, for the last panel, infection with 4 distinct VOC. We show that most, but not all, of these commercially available tests performed very well (i.e., with high sensitivity) in individuals with high viral loads (*C_T_* ≤ 25) and in individuals sampled early in the course of their infection, i.e., the most contagious individuals. One limitation of the use of *C_T_* values is the assumption that the same level of viral load is associated with the same risk of infectiousness. Nevertheless, this relationship is not linear. In the context of omicron infection, it has been shown that the level of SARS-CoV-2 replication in nasal and/or nasopharyngeal epithelia can be low during the first days of infection (12). This delay in high-level replication, while replication is already active in the oropharyngeal compartment, elicits falsely negative LFT results, although the patients have a higher risk of being infectious than individuals identified later with similar viral loads.

Postmarketing evaluations of SARS-CoV-2 antigen LFTs are needed to measure the impact of the emergence of new viral variants on the capacities to diagnose these infections. Changes in the conformation of the target antigen (the nucleoprotein for the majority of LFTs and the spike protein for some of them) could affect antibody binding, thereby reducing the sensitivity of the antigen assays. Indeed, several amino acid substitutions in the nucleoprotein (e.g., M241I, A376T, and D399N) have been shown to yield false-negative results with some antigen LFTs, despite high viral loads (13, 14). In the second part of our study, which evaluated 8 antigen LFTs found to have high sensitivity and specificity in the first part, we showed that the ability of antigen LFTs to detect the presence of the alpha, beta, delta, and omicron VOC is not altered. However, low viral loads in the nasopharynx during the first days of infection, especially during omicron infection, were not detected by LFTs, in keeping with previous findings (12, 15).

In conclusion, the performance of SARS-CoV-2 antigen LFTs relative to nucleic acid testing must be evaluated independently of the manufacturers, and only tests that achieve the required levels of sensitivity and specificity should be used. Our study assessed the performance of 22 SARS-CoV-2 antigen LFTs in 4 panels containing a large number of well-characterized samples. Although the majority of LFTs tested exhibited satisfactory sensitivity and specificity, some assays yielded a high, unacceptable proportion of false-positive results, whereas others lacked sensitivity in samples containing large amounts of virus. These LFTs should not be used in practice. The sensitivity of the best-performing assays did not vary according to the VOC, including the alpha, beta, delta, and omicron variants. Other studies will be required to evaluate whether new emerging sublineages of the omicron variant and new variants, yet to emerge, will affect SARS-CoV-2 antigen LFT performance.

## MATERIALS AND METHODS

### Materials.

This retrospective diagnostic test accuracy study was conducted in the virology laboratory of Henri Mondor University Hospital (Greater Paris area). A collection of 1,157 NPSs stored at −80°C was used (Tables 1 and 4). This anonymous retrospective study protocol followed the ethical guidelines of the Declaration of Helsinki. Data collection was declared and approved by the French Committee of Data Protection and Civil Liberties (CNIL), registration number 2218612v0.

### Sample collection and testing.

The NPSs had been collected by medical staff in various VTM, including the Xpert nasopharyngeal sample collection kit (Cepheid, Sunnyvale, CA), medium for virus (Deltalab, Barcelona, Spain), and transparent media, including an in-house saline buffer (0.9% NaCl) and Greiner Bio One (Sigma-Aldrich, St. Louis, MO). Part of the suspension was initially used for routine NAAT. The remainder was stored at −80°C.

### NAAT detection of SARS-CoV-2 RNA (reference method).

During the study period, the Henri Mondor hospital virology laboratory used several platforms for SARS-CoV-2 RNA testing, based on technical developments and reagent availability. Between March and June 2020, samples were tested using an in-house RT-qPCR assay based on the Charité protocol targeting the E and RNA-dependent RNA polymerase (RdRp) genes (16) and on a commercially available RT-PCR assay targeting the E and S genes (RealStar SARS-CoV-2 RT PCR kit 1.0; Altona Diagnostics GmbH, Hamburg, Germany) (17). After September 2020, SARS-CoV-2 RNA was detected by means of commercially available molecular biology technologies, including TMA (Aptima SARS CoV-2 on the Panther system; Hologic, Marlborough, MA) or RT-PCR (Alinity m; Abbott Molecular, Des Plaines, IL). Samples found to be RNA positive in TMA were retested by RT-PCR (TaqPath COVID-19 RT-PCR kit; Thermo Fisher, Pleasanton, CA) to measure *C_T_* values. Samples with *C_T_* values above 40 were considered negative.

### NAAT detection of other respiratory pathogens.

In NPSs collected between April 2018 and August 2019, respiratory viruses and bacteria were sought by means of a BioFire respiratory panel 2 multiplex RT-PCR assay (bioMérieux, Marcy-l’Étoile, France), according to the manufacturer’s instructions. This assay targets 16 viruses (adenovirus, coronaviruses 229E, HKU1, NL63, and OC43, Middle East respiratory syndrome CoV [MERS-CoV], SARS-CoV-2, metapneumovirus, rhinovirus/enterovirus, influenza A and B viruses, parainfluenza viruses 1 to 4, and respiratory syncytial virus) and 4 bacteria (Bordetella parapertussis, Bordetella pertussis, Chlamydia pneumoniae, and Mycoplasma pneumoniae) that commonly cause upper and lower respiratory tract infections.

### SARS-CoV-2 antigen detection by LFTs.

For antigen extraction, 80 to 350 μL of VTM (depending on the LFT) was added to each extraction buffer and mixed. Fifty to 100 μL of the mixture was added to the sample port of the antigen assay, according to the manufacturer’s instructions.

For the 20 nonautomatic reading assays, the result was read visually after 10 to 30 min. Any shade of color in the test line region was considered positive. All tests were independently read by two trained staff members. In case of discrepancy, an additional reading was performed by a third staff member. For the 2 automatic reading assays, the result was provided in 15 min or 30 min. Reading was performed twice and redone by the device in case of a discrepancy. Technicians were blind to the sample status (positive or negative for SARS-CoV-2 RNA).

### Identification of SARS-CoV-2 VOC by full-length genome sequencing.

A subset of SARS-CoV-2 RNA-positive samples collected between January 2021 and January 2022 was used to evaluate the performance of LFTs against different VOC. For VOC characterization, full-length SARS-CoV-2 genomes were sequenced by means of next-generation sequencing. Briefly, viral RNA was extracted using a NucliSENS easyMAG kit on an EMAG device (bioMérieux, Marcy-l’Étoile, France). Sequencing was performed with the Illumina COVIDSeq test (Illumina, San Diego, CA), which uses 98-target multiplex amplifications along the full SARS-CoV-2 genome. Libraries were sequenced with a NextSeq 500/550 high-output kit v2.5 (75 cycles) on a NextSeq 500 device (Illumina). The sequences were demultiplexed and assembled as full-length genomes by means of the DRAGEN COVIDSeq test pipeline on a local DRAGEN server with v1.2.2 software (Illumina). Lineages and clades were interpreted using the Lineage Assigner web application (Centre for Genomic Pathogen Surveillance, Cambridge, United Kingdom) and the Nextclade web application (Nextstrain Project, University of Basel, Basel, Switzerland) and then submitted to the GISAID international database (https://www.gisaid.org).

### Statistical analysis.

Analysis of LFT performance in part 1 of the study was conducted using RT-PCR results as the reference. Sensitivity and specificity are shown with their 95% confidence intervals calculated by means of the exact method. All qualitative data were expressed as raw numbers (percentages or *n*/*N* ratios), and statistical analyses were run with Stata 15 (StataCorp LP, College Station, TX) or with routines written in R v.3.6.1. *P* values of <0.05 were considered statistically significant.

In part 2 of the study, because the number of samples in each group of VOC was relatively small, we used a double embedded bootstrap statistical approach: for each group of variants; for each selected test, the first bootstrapping step consisted of randomly resampling positive samples with their replacement (1,000 iterations) performed for each *C_T_* stratification (Table 4). A second bootstrapping step was then applied to each iteration.

## References

[1] Fourati S, Langendorf C, Audureau E, Challine D, Michel J, Soulier A, Ahnou N, Désveaux I, Picard O, Ortonne V, Gourgeon A, Mills C, Hémery F, Rieux C, Pawlotsky J-M, Malou N, Chevaliez S. 2021. Performance of six rapid diagnostic tests for SARS-CoV-2 antigen detection and implications for practical use. J Clin Virol 142:104930. doi:10.1016/j.jcv.2021.104930. 34390929 PMC8310570

[2] Parvu V, Gary DS, Mann J, Lin Y-C, Mills D, Cooper L, Andrews JC, Manabe YC, Pekosz A, Cooper CK. 2021. Factors that influence the reported sensitivity of rapid antigen testing for SARS-CoV-2. Front Microbiol 12:714242. doi:10.3389/fmicb.2021.714242. 34675892 PMC8524138

[3] Fourati S, Soulier A, Gourgeon A, Khouider S, Langlois C, Galbin A, Bouter AL, Rodriguez C, Joanny M, Dublineau A, Challine D, Bouvier-Alias M, Chevaliez S, Audureau É, Pawlotsky J-M. 2022. Performance of a high-throughput, automated enzyme immunoassay for the detection of SARS-CoV-2 antigen, including in viral “variants of concern”: implications for clinical use. J Clin Virol 146:105048. doi:10.1016/j.jcv.2021.105048. 34863056 PMC8628626

[4] European Commission Directorate-General for Health and Food Safety, Public health, Health Security. 2022. EU health preparedness: a common list of COVID-19 rapid antigen tests; A common standardised set of data to be included in COVID-19 test result certificates and A common list of COVID-19 laboratory based antigenic assays. https://health.ec.europa.eu/system/files/2022-06/covid-19_rat_common-list_en_0.pdf.

[5] World Health Organization. Antigen-detection in the diagnosis of SARS-CoV-2 infection. 2021. https://www.who.int/publications-detail-redirect/antigen-detection-in-the-diagnosis-of-sars-cov-2infection-using-rapid-immunoassays. Retrieved 10 November 2021.

[6] Bullard J, Dust K, Funk D, Strong JE, Alexander D, Garnett L, Boodman C, Bello A, Hedley A, Schiffman Z, Doan K, Bastien N, Li Y, Van Caeseele PG, Poliquin G. 2020. Predicting infectious severe acute respiratory syndrome coronavirus 2 from diagnostic samples. Clin Infect Dis 71:2663–2666. doi:10.1093/cid/ciaa638. 32442256 PMC7314198

[7] Dinnes J, Deeks JJ, Berhane S, Taylor M, Adriano A, Davenport C, Dittrich S, Emperador D, Takwoingi Y, Cunningham J, Beese S, Domen J, Dretzke J, Ferrante di Ruffano L, Harris IM, Price MJ, Taylor-Phillips S, Hooft L, Leeflang MM, McInnes MD, Spijker R, Van den Bruel A, Cochrane COVID-19 Diagnostic Test Accuracy Group. 2021. Rapid, point-of-care antigen and molecular-based tests for diagnosis of SARS-CoV-2 infection. Cochrane Database Syst Rev 3:CD013705. doi:10.1002/14651858.CD013705.pub2. 33760236 PMC8078597

[8] Brümmer LE, Katzenschlager S, Gaeddert M, Erdmann C, Schmitz S, Bota M, Grilli M, Larmann J, Weigand MA, Pollock NR, Macé A, Carmona S, Ongarello S, Sacks JA, Denkinger CM. 2021. Accuracy of novel antigen rapid diagnostics for SARS-CoV-2: a living systematic review and meta-analysis. PLoS Med 18:e1003735. doi:10.1371/journal.pmed.1003735. 34383750 PMC8389849

[9] Deeks JJ, Singanayagam A, Houston H, Sitch AJ, Hakki S, Dunning J, Lalvani A. 2022. SARS-CoV-2 antigen lateral flow tests for detecting infectious people: linked data analysis. BMJ 376:e066871. doi:10.1136/bmj-2021-066871. 35197270 PMC8864475

[10] Corman VM, Haage VC, Bleicker T, Schmidt ML, Mühlemann B, Zuchowski M, Jo WK, Tscheak P, Möncke-Buchner E, Müller MA, Krumbholz A, Drexler JF, Drosten C. 2021. Comparison of seven commercial SARS-CoV-2 rapid point-of-care antigen tests: a single-centre laboratory evaluation study. Lancet Microbe 2:e311–e319. doi:10.1016/S2666-5247(21)00056-2. 33846704 PMC8026170

[11] Kohmer N, Toptan T, Pallas C, Karaca O, Pfeiffer A, Westhaus S, Widera M, Berger A, Hoehl S, Kammel M, Ciesek S, Rabenau HF. 2021. The comparative clinical performance of four SARS-CoV-2 rapid antigen tests and their correlation to infectivity in vitro. JCM 10:328. doi:10.3390/jcm10020328. 33477365 PMC7830733

[12] Adamson B, Sikka R, Wyllie AL, Premsrirut P. 2022. Discordant SARS-CoV-2 PCR and rapid antigen test results when infectious: a December 2021 cccupational case series. medRxiv. https://www.medrxiv.org/content/10.1101/2022.01.04.22268770v1.

[13] Del Vecchio C, Brancaccio G, Brazzale AR, Lavezzo E, Onelia F, Franchin E, Manuto L, Bianca F, Cianci V, Cattelan A, Toppo S, Crisanti A. 2021. Emergence of N antigen SARS-CoV-2 genetic variants escaping detection of antigenic tests. medRxiv. https://www.medrxiv.org/content/10.1101/2021.03.25.21253802v1.

[14] Bourassa L, Perchetti GA, Phung Q, Lin MJ, Mills MG, Roychoudhury P, Harmon KG, Reed JC, Greninger AL. 2021. A SARS-CoV-2 nucleocapsid variant that affects antigen test performance. J Clin Virol 141:104900. doi:10.1016/j.jcv.2021.104900. 34171548 PMC8219478

[15] Soni A, Herbert C, Filippaios A, Broach J, Colubri A, Fahey N, Woods K, Nanavati J, Wright C, Orwig T, Gilliam K, Kheterpal V, Suvarna T, Nowak C, Schrader S, Lin H, O’Connor L, Pretz C, Ayturk D, Orvek E, Flahive J, Lazar P, Shi Q, Achenbach C, Murphy R, Robinson M, Gibson L, Stamegna P, Hafer N, Luzuriaga K, Barton B, Heetderks W, Manabe YC, McManus D, RADx Clinical Studies Core team, Test Us AT Home investigators. 2022. Comparison of rapid antigen tests’ performance between delta (B.1.61.7; AY.X) and omicron (B.1.1.529; BA1) variants of SARS-CoV-2: secondary analysis from a serial home self-testing study. medRxiv. https://www.medrxiv.org/content/10.1101/2022.02.27.22271090v2. 10.7326/M22-0760 PMC9578286 36215709

[16] Corman VM, Landt O, Kaiser M, Molenkamp R, Meijer A, Chu DK, Bleicker T, Brünink S, Schneider J, Schmidt ML, Mulders DG, Haagmans BL, van der Veer B, van den Brink S, Wijsman L, Goderski G, Romette J-L, Ellis J, Zambon M, Peiris M, Goossens H, Reusken C, Koopmans MP, Drosten C. 2020. Detection of 2019 novel coronavirus (2019-nCoV) by real-time RT-PCR. Euro Surveill 25:2000045. doi:10.2807/1560-7917.ES.2020.25.3.2000045. 31992387 PMC6988269

[17] Visseaux B, Le Hingrat Q, Collin G, Ferré V, Storto A, Ichou H, Bouzid D, Poey N, de Montmollin E, Descamps D, Houhou-Fidouh N. 2020. Evaluation of the RealStar SARS-CoV-2 RT-PCR kit RUO performances and limit of detection. J Clin Virol 129:104520. doi:10.1016/j.jcv.2020.104520. 32652476 PMC7323686

