# Supplementary material for: Performance of 22 Rapid Lateral Flow Tests for SARS-CoV-2 Antigen Detection and Influence of “Variants of Concern”: Implications for Clinical Use
Source: Microbiol Spectr. 2022 Jul 12;10(4):e01157-22. doi: 10.1128/spectrum.01157-22 (PMC9430704; doi:10.1128/spectrum.01157-22)
Supplement: Supplemental file 1 — Tables S1 to S3 and Fig. S1. Download spectrum.01157-22-s0001.pdf, PDF file, 0.5 MB [file spectrum.01157-22-s0001.pdf]

Supplementary Table 1. SARS-CoV-2 antigen LFTs evaluated in the study, with the abbreviations used to designate them in the text, tables and figures. NB: all LFTs assessed with Cohort/Panels 2 and 3 used visual reading. * NM = not mentioned

		Test	Manufacturer	Abbreviation	Targeted antigen	Volume of transport buffer (µL)	Number of drops applied to the device	Time to read
**Comparator**		COVID-VIRO® Antigen Rapid Test	AAZ	AAZ	N protein	100 µL	3 drops	15 min
**Panel 1**	Visual reading	CerTest SARS-CoV-2 Card Test	CerTest Biotec	CerTest	N protein	100 µL	3 drops	10 min
		GenBody COVID-19 Ag	GenBody Inc.	GenBody	N protein	100 µL	4 drops	20 min
		NADAL COVID-19 Ag test	Nal von Minden	Nadal	N protein	100 µL	2 drops	15 min
		Rapid Test Antigen GENSURE COVID-19	Gensure	GenSure	N protein	100 µL	3 drops	15 min
		AMP Rapid Test SARS-CoV-2 Ag	AMP Diagnostics	AMP	N protein	100 µL	4 drops	15 min
		QuickProfile™ COVID-19 Antigen Test	LumiQuick Diagnostics	Quick Profile	N protein	100 µL	Strip immersion	10 min
		Novel Coronavirus (COVID-19) Antigen Test Kit	Medakit	Novel	N protein	100 µL	3 drops	15 min
		Toda Coronadiag Ag	Toda Pharma	Toda Pharma	N protein	100 µL	2 drops	15 min
	Automatic reading	SOFIA SARS Antigen FIA	Quidel	Sofia	N protein	250 µL	4 drops	30 min
		WANTAI SARS-Cov-2 Ag Rapid test	Eurobio Scientific	Wantai	N protein	80 µL	70 µL	15 min
**Panel 2**		SARS-CoV-2 Antigen Rapid Detection Kit	Genomic vision	Genomic Vision	NM*	100 µL	3 drops	15 min

	RealityTech Antigen Test COVID 19	Fasual Care	Fasual Care	NM*	100 µL	3 drops	15 min
	COVID-19 ANTIGEN RAPID TEST	Servibio	ServiBio	NM*	100 µL	3 drops	15 min
	HUMASIS ONE STEP COVID 19 AG TEST	Eurobio Scientific	Humasis	N protein<br>S protein	100 µL	3 drops	15 min
	BSD-0500333-25- COVID19 Speed-Antigen Test	Biospeedia	BioSpeedia	NM*	100 µL	2 drops	20 min
	SARS-CoV-2 Spike Colloidal Gold Chromatographic assay	RBiopharm	R Biopharm	S protein	100 µL	2 drops	15 min
	Test antigénique rapide CLINITEST COVID 19	Siemens Healthcare	Siemens	N protein	100 µL	4 drops	15 min
	NG-Test COVID19	NG-Biotech	NG Biotech 2	N protein	120 µL	3 drops	15 min
	INDICAID COVID-19 RAPID ANTIGEN TEST	Medisur	Medisur	N protein	100 µL	3 drops	20 min
**Panel 3**	PCL TEST COVID AG	Tanit Care	Tanit Care	N protein	100 µL	5 drops	10 min
	BIOSENSOR Standard F COVID-19 Ag FIA	Orgentec	Orgentec	N protein	350 µL	4 drops	30 min

Supplementary Figure 1. Ct distribution in the 3 SARS-CoV-2 RNA-positive panels described in **Table 1**. NB: The Sofia and Wantai tests were studied on subsets of the original panel, including 133 and 118 samples, respectively, collected on a transparent VTM.

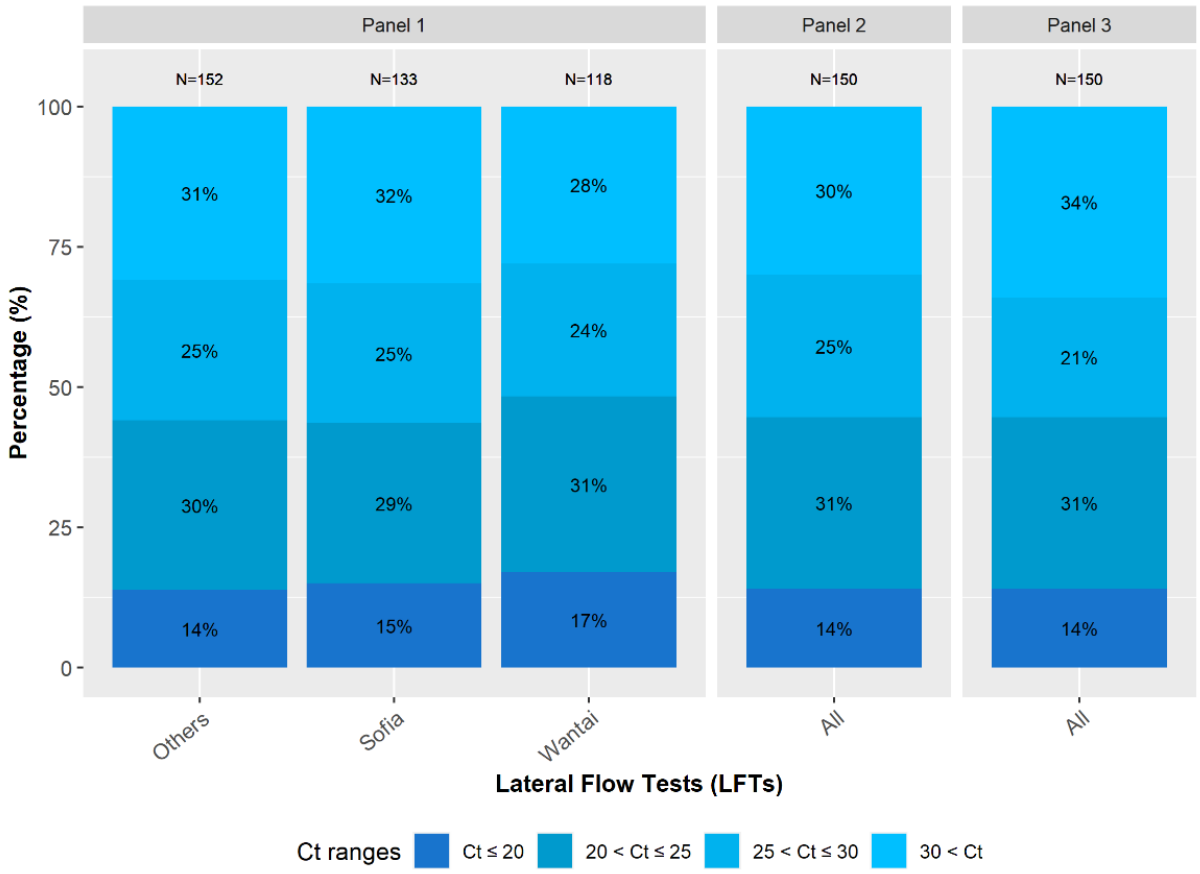

Supplementary Table 2. Rates of success of the different RDTs and invalid results in SARS-CoV-2 RNA-positive and negative samples. Non-presented RDT get a 100% rate of success.

	Tests	Rate of success (%)	Number (%) of invalid results	
			SARS-CoV-2 RNA-positive	SARS-CoV-2 RNA-negative
**Panel 1**	GenBody[1]	96.0%	8 (5.3%)	4 (2.7%)
	Nadal	98.7%	-	4 (2.7%)
	GenSure	99.3%	2 (1.3%)	-
	AMP	99.3%	1 (0.7%)	1 (0.7%)
	Quick Profile	99.7%	1 (0.7%)	-
	Novel[1]	99.0%	2 (1.3%)	1 (0.7%)
	Toda Pharma	99.3%	2 (1.4%)	-
	Sofia	99.3%	1 (0.7%)	-
**Panel 2**	Fasual Care	99.3%	1 (0.7%)	1 (0.8%)
	Humasis	99.6%	-	1 (0.8%)
	R Biopharm	99.3%	1 (0.7%)	-
**Panel 3**	Orgentec	99.7%	1 (0.7%)	-
			**Total: N=20**	**Total: N=12**

[1] one invalid result from the same sample

Supplementary Table 3. Other respiratory viruses found in SARS-CoV-2 RNA-negative samples used for specificity evaluations containing at least one respiratory virus.

**Respiratory Viruses**	**Panel 1 (n=76)**	**Panel 2 (n=25)**	**Panel 3 (n=26)**
Adenovirus	6	1	1
Metapneumovirus	7	2	2
Rhinovirus/Enterovirus	7	3	3
Respiratory syncytial virus	7	2	2
Influenza A virus	24	2	3
Influenza B virus	5	1	1
Parainfluenza virus 1	2	-	-
Parainfluenza virus 2	2	-	-
Parainfluenza virus 3	2	3	3
Parainfluenza virus 4	2	3	3
Coronavirus 229E	3	2	2
Coronavirus HKU1	3	2	2
Coronavirus NL63	3	2	2
Coronavirus OC43	3	2	2
